# Lower Successful Quit Rate of Menthol Tobacco Users in a Tobacco Cessation Program: An Explanatory Analysis in Search of Potential Mechanisms

**DOI:** 10.31586/gjcd.2025.1279

**Published:** 2025-02-26

**Authors:** Payam Sheikhattari, Rifath Ara Alam Barsha, Shervin Assari

**Affiliations:** 1 Prevention Sciences Research Center, Morgan State University, Baltimore, MD, USA; 2 School of Community Health & Policy, Morgan State University, Baltimore, MD, USA; 3 Johns Hopkins University, Baltimore, MD, USA; 4 University of Mississippi Medical Center, MS, USA; 5 Charles R Drew University of Medicine and Science, Los Angeles, CA, USA

**Keywords:** Menthol Tobacco, Smoking Cessation, Randomized Controlled Trial, Intervention Effectiveness, Quit Rates

## Abstract

**Background:**

Menthol-flavored tobacco products are disproportionately used in low-income African American communities, a result of decades of targeted marketing and systemic inequities. Menthol use has been associated with lower quit rates, often compounded by factors such as lower trust in healthcare systems, reduced access to cessation programs, and other structural barriers. Despite this, few studies have systematically examined the explanatory mechanisms that might clarify why menthol-flavored tobacco is linked to poorer cessation outcomes among participants in tobacco cessation programs.

**Aims:**

This study aimed to investigate the potential mechanisms by which menthol tobacco use is associated with lower quit rates across three types of smoking cessation interventions.

**Methods:**

Participants were randomized into one of three smoking cessation interventions: in-person (CEASE), self-help, or online/hybrid programs. Smoking abstinence was assessed three months post-intervention as the primary outcome. Secondary analyses explored whether demographic, socioeconomic, or behavioral factors mediated the association between menthol use and quit rates across the intervention arms.

**Results:**

Menthol tobacco use was significantly associated with lower quit rates (p < 0.01). This association was not explained by demographic, socioeconomic, health, or addiction-related factors. While menthol use was associated with lower education and employment levels, demographic characteristics, physical or mental health, or addiction did not explain the effect of menthol on tobacco cessation. These findings suggest that the lower quit rates observed among menthol users cannot be attributed to any third factors assessed in this study.

**Conclusions:**

Menthol tobacco use independently predicts lower quit rates, and the mechanisms behind this disparity remain unclear. The consistent findings across different intervention types highlight the need for further research to uncover the underlying pathways and to design targeted strategies to improve cessation outcomes for menthol users.

## 1. Introduction

Menthol-flavored tobacco products present a significant challenge to public health, particularly due to their potential link to higher addiction and lower quit rates [1,2]. This issue is especially pronounced in low-income African American communities [2–4], where menthol products are disproportionately marketed and consumed. Decades of predatory marketing by tobacco companies have targeted these communities through strategies such as price discounts, culturally tailored advertising, and increased retail availability [3–5]. These tactics have led to the high prevalence of menthol tobacco use among African Americans, a population already burdened by systemic inequities in healthcare access and cessation resources. The combination of aggressive marketing and structural barriers underscores the urgent need to understand the mechanisms that drive the lower quit rates among menthol users in these communities [2].

One potential explanation for menthol users’ lower quit rates is the role of addiction severity. Menthol, a compound derived from mint, has unique properties that may enhance nicotine’s addictive potential [1,6,7]. Neurobiological and animal studies suggest that menthol amplifies nicotine’s rewarding effects by modulating pathways in the brain associated with addiction. For example, menthol interacts with nicotinic acetylcholine receptors, increasing dopamine release in brain regions associated with reward and dependence [6,8,9]. Additionally, animal studies have shown that menthol enhances nicotine self-administration and alleviates withdrawal symptoms, suggesting that the pairing of menthol with nicotine may result in a stronger addiction profile [10,11]. These findings suggest that menthol may act as more than just a flavoring agent, potentially altering physiological and behavioral responses to nicotine in ways that complicate cessation efforts.

Another plausible explanation involves socioeconomic status [12,13]. If menthol users differ significantly from non-menthol users in socioeconomic status —given economic resources’ established role in influencing quit rates [14–16]—these differences may partly explain the observed disparities. Similarly, differences in physical [17,18] and mental health [19,20] profiles between menthol and non-menthol users could act as additional mechanisms. Demographic factors such as age and sex, which are known predictors of cessation success, might also contribute to these differences if they correlate with menthol use. Finally, patterns of tobacco use, including pack-years, product type, and quitting history, may further clarify whether the association between menthol and lower quit rates is mediated by these factors.

Despite the insights provided by previous research, the exact mechanisms through which menthol influences addiction severity and cessation outcomes remain poorly understood. This study seeks to examine the interplay between addiction severity, demographic characteristics, socioeconomic status indicators, physical and mental health, and tobacco use patterns to elucidate why menthol use is associated with lower quit rates. By addressing these questions, we aim to inform more effective and equitable tobacco cessation strategies tailored to the needs of menthol users, particularly those in low-income African American communities.

## 2. Methods

### 2.1. Study Design and Setting

This study utilized a randomized cluster trial design to evaluate smoking cessation interventions across three Baltimore City communities [21–27]. Communities were randomly assigned to one of three intervention arms: (1) in-person intervention, (2) virtual/hybrid intervention, and (3) self-help/control group. Randomization was conducted in a blinded manner during a steering committee meeting to reduce potential bias. The selected communities—Oldtown/Middle East, Waverly, and Southwest (Poppleton/The Terraces/Hollins Market/Washington Village/Pigtown)—were chosen based on comparable sociodemographic characteristics and access to shared community resources, such as schools, churches, and healthcare centers. Initially, the virtual intervention was implemented as a fully remote program; however, logistical challenges necessitated a transition to a hybrid model. Recruitment boundaries were expanded to neighboring areas to enhance participant enrollment. Participants outside the original community boundaries were randomized by site.

### 2.2. Ethical Considerations

The study received approval from Morgan State University’s Institutional Review Board (IRB #19/06–0082). Written informed consent was obtained from all participants prior to enrollment. To protect confidentiality, participants were assigned unique identification numbers that were stored separately from personal information.

### 2.3. Study Participants

Eligibility criteria for participation in the Communities Engaged and Advocating for a Smoke-free Environment (CEASE) [21–27] Digital smoking cessation program included: (1) age 21 years or older, (2) current smoker consuming three or more cigarettes daily, (3) willingness to quit smoking, and (4) provision of informed consent. Participants in the fully virtual intervention required reliable access to a compatible device (e.g., desktop, laptop, tablet) and consistent internet or cellular connectivity. This requirement was waived for hybrid sessions conducted in person. Individuals unable to provide informed consent due to health conditions were excluded from the study.

### 2.4. Intervention

Nine peer motivators, comprising former smokers and individuals with personal connections to tobacco use (e.g., family history of smoking or tobacco-related illness), were trained to facilitate smoking cessation classes. Training included a three-day workshop covering the CEASE Digital curriculum, digital platform integration, research ethics, study protocols, data management, and group facilitation techniques. Peer motivators also assisted in recruiting participants through outreach efforts, including flyers, social media announcements, referrals, word-of-mouth, and community surveys.

Smoking cessation sessions were conducted in community spaces, including public housing sites, churches, and senior residences. Recruitment occurred between April 2022 and September 2023. The CEASE Digital curriculum, co-developed with community stakeholders, was a seven-week program tailored to in-person and virtual/hybrid formats. In-person participants used printed materials, including the CEASE Today Tobacco Cessation Manual. The virtual/hybrid group accessed the program through an online platform mirroring the manual’s content, with virtual materials provided by peer motivators. Fully virtual sessions were conducted via Zoom, while hybrid sessions occurred in person.

All sessions followed a structured timeline: informed consent (week 1), orientation and technology training (week 2), motivation and preparation to quit (weeks 3–4), quitting strategies (weeks 5–6), and relapse prevention (week 7). Sessions were two hours weekly, facilitated by two peer motivators per cohort. Participants in the self-help/control group received existing cessation services, including a one-hour motivational session, self-help materials, and referrals to local tobacco cessation resources.

### 2.5. Questionnaires

Baseline surveys collected data on demographics, smoking history, physical and mental health, social support, and other variables. Participants in the in-person and virtual/hybrid groups were followed three months post-intervention, completing a follow-up survey to report smoking status. The self-help/control group completed follow-up surveys five months post-enrollment.

### 2.6. Measures

- **Outcome Variable:** Smoking cessation at follow-up was the primary outcome, measured as a binary variable (0 = did not quit, 1 = quit).
- **Explanatory variables:** Explanatory variables included age, gender, race, intervention arm, and nicotine dependence (measured by the Fagerström Test for Nicotine Dependence). Additional variables such as educational attainment, family income, marital status, general health, substance use, depression (PHQ-2), stress (PSS-4), and social support (DUFSSQ) were included in descriptive analyses.

### 2.7. Statistical Analysis

Descriptive statistics were computed for all variables, with means and proportions summarized by study arm. Bivariate analyses, including chi-square tests for categorical variables and independent samples t-tests for continuous variables, assessed differences across arms. Logistic regression models examined predictors of response to explain the effects of menthol on smoking cessation status, with results reported as odds ratios (ORs), 95% confidence intervals (CIs), and p-values. All analyses were conducted using Stata 15.0, with statistical significance set at p < 0.05.

## 3. Results

Table 1 presents the descriptive results of the study both overall and stratified by menthol/multiple-flavor use. The majority of participants were over 50 years old (78.4%), women (50.5%), and Black Americans (82.8%). The mean (SD) nicotine addiction score at baseline was 4.4 (2.0), and the mean (SD) depression score was 4.1 (1.0).

While 38.9% of menthol/multiple-flavor non-users reported quitting smoking, only 18.5% of menthol/multiple-flavor users reported quitting. The mean (SD) number of cardiometabolic risk conditions was 0.7 (1.0) for menthol/multiple-flavor non-users and 1.1 (0.9) for menthol/multiple-flavor users.

Table 2 presents the bivariate correlations among the study variables. Quit smoking (r = −0.14, p < 0.05) and other tobacco products use (r = −0.17, p < 0.05) showed a significant negative correlation with menthol/multiple-flavor use. No other variables were significantly correlated with menthol/multiple-flavor use.

The results of the multivariable logistic regression analysis are presented in Table 3. Individuals receiving in-person interventions showed significantly higher odds of quitting (AOR = 3.81, *p* < 0.05). Menthol/multiple-flavor tobacco users were less likely to quit compared to non-users (AOR = 0.14, *p* < 0.01). Women had lower odds of quitting compared to men (AOR = 0.33, *p* < 0.01). Participants with a higher number of cardiometabolic risk conditions were more likely to quit smoking (AOR = 1.73, *p* < 0.05), while individuals with higher levels of depression had lower odds of quitting (AOR = 0.56, *p* < 0.05).

## 4. Discussion

This study aimed to explore why menthol tobacco use is associated with lower quit rates in smoking cessation programs, focusing on potential explanatory mechanisms. Using a three-arm randomized trial of in-person (CEASE)[21–28], self-help, and online/hybrid interventions, we investigated whether demographic, socioeconomic, health, or addiction-related factors could explain the observed disparity in quit rates among menthol users. Despite comprehensive analyses, we found that menthol tobacco use independently predicted lower quit rates, with no evidence that these factors mediated the association.

Our analyses revealed no significant associations between menthol use and demographic characteristics such as age, gender, or race. While menthol tobacco use is disproportionately prevalent in certain demographic groups, particularly among African Americans, our findings suggest that these differences do not account for the observed disparities in quit rates. This indicates that the lower cessation success among menthol users may not be solely a result of demographic composition but rather due to other, unmeasured factors.

Menthol use was associated with lower education and employment levels in our sample, which aligns with prior research indicating that menthol tobacco products are disproportionately marketed in low-income communities. However, these socioeconomic differences did not explain the lower quit rates among menthol users. This finding underscores the unique challenges faced by menthol users that persist beyond the influence of socioeconomic status, suggesting that other mechanisms may be at play.

No significant associations were found between menthol use and physical or mental health indicators, such as stress or depression. These findings challenge the hypothesis that menthol users are less likely to quit because they have more comorbidities, have more depression, have more stress, or have less social support. Other physiological or behavioral pathways may contribute to their low quit rates of menthol users.

The role of addiction severity, often implicated in cessation difficulties, also failed to emerge as a significant mechanism in our study. Contrary to expectations based on neurobiological and animal research, menthol users did not exhibit higher scores on the Fagerström Test for Nicotine Dependence (FTND) [29–36], nor were there significant differences in individual FTND components. While these findings challenge the hypothesis that addiction severity directly mediates the relationship between menthol use and lower quit rates, they do not rule out the possibility that menthol interacts with nicotine in ways that are not fully captured by traditional measures of dependence. The potential neurobiological mechanisms underlying menthol’s influence on addiction remain an important area for further exploration. Research has demonstrated that menthol enhances nicotine’s rewarding effects by modulating dopaminergic activity in brain regions associated with reward and dependence. Additionally, menthol may reduce the aversive sensations associated with smoking, making tobacco use more appealing and reinforcing. These neurochemical interactions could create a unique addiction profile for menthol users, making them more resistant to traditional cessation interventions.

Menthol is widely used in tobacco products for its cooling and anesthetic properties, which reduce the harshness of smoking and increase its appeal. These properties have been exploited through targeted marketing campaigns in low-income and African American communities, leading to disproportionately high menthol use in these populations. Research suggests that menthol enhances nicotine’s addictive potential by modulating reward pathways in the brain, which may contribute to the lower quit rates observed in menthol users.

The disproportionate use of menthol tobacco among low-income African American communities is largely a consequence of predatory marketing by tobacco companies. Policies aimed at banning menthol products could significantly reduce the appeal and availability of these products, thereby mitigating the disparities in quit rates. Furthermore, stricter regulations on targeted advertising and retail density in low-SES areas could decrease the accessibility of menthol products and reduce initiation rates. Coupled with broader tobacco control efforts, these policies could help address the systemic inequities that have made menthol a significant driver of health disparities in these communities.

### 4.1. Implications

This study provides additional evidence supporting the argument that menthol and flavored tobacco products not only increase their appeal among youth and racial minorities but also make quitting more difficult. Therefore, policies banning flavored tobacco products could play a crucial role in mitigating these challenges. In addition, our findings highlight the unique challenges faced by menthol users in cessation programs, emphasizing the need for tailored interventions. Standard approaches that assume uniform efficacy across all smokers may be insufficient for this subgroup. Public health strategies must account for the specific barriers faced by menthol users, including potential neurobiological and sensory mechanisms that may reinforce addiction.

Clinicians and quit-line operators should provide additional support to menthol users, recognizing their heightened risk of cessation failure. Tailored interventions, such as extended counseling, culturally appropriate support, and strategies to mitigate the sensory and psychological appeal of menthol, may improve outcomes. Training cessation providers to address the unique challenges of menthol users could also enhance the effectiveness of these programs.

### 4.2. Limitations

This study has several limitations. First, our assessment was limited to three months post-intervention, and longer follow-up periods may provide additional insights into quit trajectories. Second, while we included a comprehensive set of variables, other unmeasured factors, such as neurobiological mechanisms or environmental influences, may play a role. Third, our findings may not generalize to populations outside of the study sample, particularly given the overrepresentation of menthol use in certain demographic groups.

### 4.3. Future Research

Future studies should explore the neurobiological mechanisms underlying the interaction between menthol and nicotine, including how menthol affects reward processing and withdrawal symptoms. Qualitative research capturing the lived experiences of menthol users may also provide valuable insights into behavioral and psychosocial barriers to cessation. Finally, intervention studies should evaluate the efficacy of tailored cessation approaches designed specifically for menthol users, including pharmacological and behavioral strategies.

## 5. Conclusions

Menthol tobacco use is associated with lower quit rates in cessation programs, and this disparity cannot be explained by demographic, socioeconomic, health, or addiction-related factors. These findings underscore the urgent need for targeted research and tailored interventions to address the challenges faced by menthol users. By understanding and addressing the unique barriers to cessation in this population, we can advance health equity and reduce tobacco-related disparities.

## Figures and Tables

**Table 1.** Descriptive Statistics of the Study Sample - Overall and by Menthol/Multiple Flavor Use

Variables	Menthol/Multiple Flavor Use			Overall (n=232)
	No (n=18)	Yes (n=206)	Missing (n=8)	
Quit Smoking				
No	11 (61.1)	168 (81.5)	7 (87.50)	186 (80.17)
Yes	7 (38.9)	38 (18.5)	1 (12.50)	46 (19.83)
Study Arm				
In-person	7 (38.9)	71 (34.5)	1 (12.50)	79 (34.0)
Virtual/Hybrid	4 (22.2)	82 (39.8)	4 (50.00)	90 (38.8)
Self-help	7 (38.9)	53 (25.7)	3 (37.50)	63 (27.2)
Age				
50 Years or Less	5 (27.8)	41 (19.9)	1 (12.50)	47 (20.3)
More than 50 Years	12 (66.7)	163 (79.1)	7 (87.50)	182 (78.4)
Missing	1 (5.6)	2 (1.0)	-	3 (1.3)
Gender				
Men	6 (33.3)	96 (46.6)	5 (62.50)	107 (46.1)
Women	10 (55.6)	104 (50.5)	3 (37.50)	117 (50.4)
Missing	2 (11.1)	6 (2.9)	-	8 (3.5)
Race				
Black	12 (66.7)	172 (83.5)	8 (100.0)	192 (82.8)
White	5 (27.8)	22 (10.7)	-	27 (11.6)
Other/Multiple	-	8 (3.9)	-	8 (3.4)
Missing	1 (5.6)	4 (1.9)	-	5 (2.2)
Educational Attainment				
Some High School or Less	3 (16.7)	57 (27.7)	4 (50.0)	64 (27.6)
Graduated from High School/GED	5 (27.8)	76 (36.9)	1 (12.5)	82 (35.3)
Some College	4 (22.2)	48 (23.3)	2 (25.0)	54 (23.3)
Bachelor or More	5 (27.8)	22 (10.7)	1 (12.5)	28 (12.1)
Missing	1 (5.5)	3 (1.4)	-	4 (1.7)
Other Tobacco Product Use**				
No	10 (55.6)	165 (80.1)	5 (62.5)	180 (77.6)
Yes	8 (44.4)	39 (18.9)	2 (25.0)	49 (21.1)
Missing	-	2 (1.0)	1 (12.5)	3 (1.3)
	Mean (SD)	Mean (SD)		Mean (SD)
Nicotine Addiction (at Baseline)	4.4 (2.1)	4.4 (2.0)		4.4 (2.0)
Number of Cardiometabolic Risk Conditions	0.7 (1.0)	1.1 (0.9)		1.1 (1.0)
Depression	1.5 (1.7)	1.3 (1.7)		1.3 (1.7)

** P < 0.01

**Table 2.** Correlation Matrix of the Study Variables Correlation Matrix of the Study Variables

	1	2	3	4	5	6	7	8	9
1.Menthol/Multiple Flavor Use	1.00								
2.Quit Smoking	−0.14*	1.00							
3.Age (Yr)	0.06	0.04	1.00						
4.Gender (Women)	−0.06	−0.11	0.06	1.00					
5. Education	−0.13	0.09	−0.01	0.05	1.00				
6. Number of Cardiometabolic Risk Conditions	0.10	0.10	0.28***	0.12	0.05	1.00			
7. Depression	−0.04	−0.14*	0.00	0.02	−0.03	0.17*	1.00		
8. Other Tobacco Products Use	−0.17*	0.0839	−0.17*	−0.08	−0.01	−0.09	0.11	1.00	
9. Nicotine Addiction (at Baseline)	0.00	−0.06	−0.01	−0.08	−0.18**	−0.01	0.22**	−0.02	1.00

Note:

*** P < 0.001,

** P < 0.01,

* P < 0.05.

**Table 3.** Logistic Regression Results

Variables	Quit Smoking	
	Unadjusted OR (95% CI)	Adjusted OR (95% CI)
Menthol/Multiple Flavor Use		
No	Ref.	Ref.
Yes	0.36* (0.13, 0.98)	0.14** (0.04, 0.55)
Study Arm		
Self-Help Group	Ref.	Ref.
In-Person Group	2.17 (0.92, 5.16)	3.81* (1.21, 12.03)
Virtual/Hybrid Group	1.30 (0.53, 3.16)	2.19 (0.67, 7.18)
Age		
50 years or Less	Ref.	Ref.
More than 50 years	1.29 (0.56, 2.98)	1.66 (0.59, 4.69)
Gender		
Men	Ref.	Ref.
Women	0.57 (0.30, 1.11)	0.33* (0.14, 0.77)
Educational Attainment		
Some High School or Less	Ref.	Ref.
Graduated from High School/GED	1.08 (0.46, 2.54)	0.84 (0.28, 2.50)
Some College	1.38 (0.55, 3.43)	1.26 (0.39, 4.13)
Bachelor or More	1.93 (0.68, 5.49)	0.97 (0.23, 4.07)
Number of Cardiometabolic Risk Conditions	1.29 (0.91, 1.84)	1.73* (1.13, 2.66)
Depression	0.77* (0.61, 0.98)	0.56** (0.39, 0.80)
Other Tobacco Product Use		
No	Ref.	Ref.
Yes	1.61 (0.77, 3.36)	2.50 (0.96, 6.47)
Nicotine Addiction (at Baseline)	0.93 (0.79, 1.09)	1.01 (0.82, 1.24)

Note: Abbreviations: 0R= Odds Ratio; CI=Confidence Interval;

** P < 0.01,

* P < 0.05.

## References

[1] Delnevo CD, Gundersen DA, Hrywna M, Echeverria SE, Steinberg MB. Smoking-cessation prevalence among US smokers of menthol versus non-menthol cigarettes. American journal of preventive medicine. 2011;41(4):357–365. 21961462 10.1016/j.amepre.2011.06.039

[2] Gandhi K, Foulds J, Steinberg M, Lu SE, Williams J. Lower quit rates among African American and Latino menthol cigarette smokers at a tobacco treatment clinic. International journal of clinical practice. 2009;63(3):360–367. 19222622 10.1111/j.1742-1241.2008.01969.x

[3] Gardiner PS. The African Americanization of menthol cigarette use in the United States. Nicotine & Tobacco Research. 2004;6(Suppl_1):S55–S65. 14982709 10.1080/14622200310001649478

[4] Henriksen L, Schleicher NC, Fortmann SP. Menthol cigarettes in black neighbourhoods: still cheaper after all these years. Tobacco control. 2022;31(e2):e211–e212. 34385403 10.1136/tobaccocontrol-2021-056758 PMC8837722

[5] Sklaroff LR. Pushing Cool: Big Tobacco, Racial Marketing, and the Untold Story of the Menthol Cigarette. Oxford University Press Oxford, UK; 2023.

[6] Henderson BJ. Linking nicotine, menthol, and brain changes. Neuroscience of nicotine. Elsevier; 2019:87–95.

[7] Zhang M, Harrison E, Biswas L, Tran T, Liu X. Menthol facilitates dopamine-releasing effect of nicotine in rat nucleus accumbens. Pharmacol Biochem Behav. Dec 2018;175:47–52. doi:10.1016/j.pbb.2018.09.004 30201386 PMC6240495

[8] Henderson BJ, Wall TR, Henley BM, Menthol alone upregulates midbrain nAChRs, alters nAChR subtype stoichiometry, alters dopamine neuron firing frequency, and prevents nicotine reward. Journal of Neuroscience. 2016;36(10):2957–2974. 26961950 10.1523/JNEUROSCI.4194-15.2016 PMC4783498

[9] Henderson BJ, Grant S, Wong BK, Menthol stereoisomers exhibit different effects on α4β2 nAChR upregulation and dopamine neuron spontaneous firing. Eneuro. 2018;5(6) 10.1523/ENEURO.0465-18.2018 PMC6325563 30627659

[10] Oz M, El Nebrisi EG, Yang K-HS, Howarth FC, Al Kury LT. Cellular and molecular targets of menthol actions. Frontiers in pharmacology. 2017;8:472. 28769802 10.3389/fphar.2017.00472 PMC5513973

[11] Kabbani N Not so Cool? Menthol’s discovered actions on the nicotinic receptor and its implications for nicotine addiction. Frontiers in pharmacology. 2013;4:95. 23898298 10.3389/fphar.2013.00095 PMC3720998

[12] Goodwin RD, Ganz O, Weinberger AH, Smith PH, Wyka K, Delnevo CD. Menthol cigarette use among adults who smoke cigarettes, 2008–2020: rapid growth and widening inequities in the United States. Nicotine and Tobacco Research. 2023;25(4):692–698. 36223889 10.1093/ntr/ntac214 PMC10216874

[13] Patel A, Hirschtick JL, Cook S, Sociodemographic patterns of exclusive and dual use of ENDS and menthol/non-menthol cigarettes among US youth (Ages 15–17) using two nationally representative surveys (2013–2017). International Journal of Environmental Research and Public Health. 2021;18(15):7781. 34360077 10.3390/ijerph18157781 PMC8345686

[14] Pisinger C, Aadahl M, Toft U, Jørgensen T. Motives to quit smoking and reasons to relapse differ by socioeconomic status. Preventive medicine. 2011;52(1):48–52. 21047525 10.1016/j.ypmed.2010.10.007

[15] Reid JL, Hammond D, Boudreau C, Fong GT, Siahpush M, collaboration I. Socioeconomic disparities in quit intentions, quit attempts, and smoking abstinence among smokers in four western countries: findings from the International Tobacco Control Four Country Survey. Nicotine & Tobacco Research. 2010;12(suppl_1):S20–S33. 20889477 10.1093/ntr/ntq051 PMC2948137

[16] Carlson S, Widome R, Fabian L, Luo X, Forster J. Barriers to quitting smoking among young adults: the role of socioeconomic status. American Journal of Health Promotion. 2018;32(2):294–300. 29214844 10.1177/0890117117696350 PMC5725277

[17] Mendiondo MS, Alexander LA, Crawford T. Health profile differences for menthol and non‐menthol smokers: findings from the national health interview survey. Addiction. 2010;105:124–140. 21059143 10.1111/j.1360-0443.2010.03202.x

[18] Jeffries O, Waldron M. The effects of menthol on exercise performance and thermal sensation: A meta-analysis. Journal of science and medicine in sport. 2019;22(6):707–715. 30554924 10.1016/j.jsams.2018.12.002

[19] Albishi FM, Albeshi SM, Alotaibi K, The effect of menthol on anxiety and related behaviors in mice. Bahrain Medical Bulletin. 2020;42(4):275–9.

[20] Cohn AM, Johnson AL, Hair E, Rath JM, Villanti AC. Menthol tobacco use is correlated with mental health symptoms in a national sample of young adults: implications for future health risks and policy recommendations. Tobacco Induced Diseases. 2016;14:1–10. 26752983 10.1186/s12971-015-0066-3 PMC4705641

[21] Apata J, Oladele A, Fahimi S, Monday-Enhanced CEASE Program for Underserved Ethnic Minorities: a Mixed-Methods Study. J Racial Ethn Health Disparities. Mar 30 2023:1–15. doi:10.1007/s40615-023-01570-0 PMC10062259 36995578

[22] Apata J, Sheikhattari P, Bleich L, Kamangar F, O’Keefe AM, Wagner FA. Addressing Tobacco Use in Underserved Communities Through a Peer-Facilitated Smoking Cessation Program. Journal of Community Health. 2019/10/01 2019;44(5):921–931. doi:10.1007/s10900-019-00635-8 30843139 PMC6708456

[23] Estreet A, Apata J, Kamangar F, Improving participants’ retention in a smoking cessation intervention using a community-based participatory research approach. International Journal of Preventive Medicine. 2017;8:106. doi:10.4103/ijpvm.IJPVM_303_17 29416835 PMC5760842

[24] Petteway RJ, Sheikhattari P, Wagner F. Toward an Intergenerational Model for Tobacco-Focused CBPR: Integrating Youth Perspectives via Photovoice. Health Promot Pract. Jan 2019;20(1):67–77. doi:10.1177/1524839918759526 29514503 PMC6119506

[25] Sheikhattari P, Apata J, Bleich L, Kamangar F, Assari S. Efficacy of a Smoking Cessation Program for Underserved Ethnic Minority Communities: Results of a Smoking Cessation Trial. Int J Public Health. 2023;68:1605739. doi:10.3389/ijph.2023.1605739 37408795 PMC10318133

[26] Sheikhattari P, Apata J, Kamangar F, Examining Smoking Cessation in a Community-Based Versus Clinic-Based Intervention Using Community-Based Participatory Research. J Community Health. 2016;41(6):1146–1152. doi:10.1007/s10900-016-0264-9 27688221 PMC5083217

[27] Sheikhattari P, Barsha RAA, Egboluche C, Foster A, Assari S. In-Person versus Virtual CEASE Smoking Cessation Interventions. Journal of biomedical and life sciences. 2024;4(2):71. 39575231 10.31586/jbls.2024.1107 PMC11580340

[28] Assari S, Alam Barsha RA, Egboluche C, Sheikhattari P. The CEASE Tobacco Cessation Controlled Trial for Low-Income Racial and Ethnic Minority Participants: Key Predictors of Success. Global Journal of Cardiovascular Diseases, 2025, 4(1), 1246. DOI: 0.31586/gjcd.2025.1246 40191287 10.31586/gjcd.2025.1246 PMC11970950

[29] Mushtaq N, Beebe LA. Psychometric Properties of Fagerström Test for Nicotine Dependence for Smokeless Tobacco Users (FTND-ST). Nicotine Tob Res. 2017 Sep 1;19(9):1095–1101. doi: 10.1093/ntr/ntx076. 28387864

[30] Mushtaq N, Beebe LA. Assessment of the Tobacco Dependence Screener Among Smokeless Tobacco Users. Nicotine Tob Res. 2016;18(5):885–91. doi: 10.1093/ntr/ntv283. 26718743

[31] Meneses-Gaya IC, Zuardi AW, Loureiro SR, Crippa JA. Psychometric properties of the Fagerström Test for Nicotine Dependence. J Bras Pneumol. 2009;35(1):73–82. English, Portuguese. doi: 10.1590/s1806-37132009000100011. 19219334

[32] Rustin TA. Assessing nicotine dependence. Am Fam Physician. 2000 Aug 1;62(3):579–84, 591–2. 10950214

[33] Ebbert JO, Patten CA, Schroeder DR. The Fagerström Test for Nicotine Dependence-Smokeless Tobacco (FTND-ST). Addict Behav. 2006;31(9):1716–21. doi: 10.1016/j.addbeh.2005.12.015. 16448783 PMC1618870

[34] de Granda-Orive JI, Pascual-Lledó JF, Asensio-Sánchez S, Solano-Reina S, García-Rueda M, Martínez-Muñiz MÁ, Lázaro-Asegurado L, Bujulbasich D, Pendino R, Luhning S, Cienfuegos-Agustín I, Jiménez-Ruiz CA. Fagerström Test and Heaviness Smoking Index. Are they Interchangeable as a Dependence Test for Nicotine? Subst Use Misuse. 2020;55(2):200–208. doi: 10.1080/10826084.2019.1660680. 31519135

[35] Etter JF, Duc TV, Perneger TV. Validity of the Fagerström test for nicotine dependence and of the Heaviness of Smoking Index among relatively light smokers. Addiction. 1999;94(2):269–81. doi: 10.1046/j.1360-0443.1999.94226910.x. 10396794

[36] Svicher A, Cosci F, Giannini M, Pistelli F, Fagerström K. Item Response Theory analysis of Fagerström Test for Cigarette Dependence. Addict Behav. 2018;77:38–46. doi: 10.1016/j.addbeh.2017.09.005. 28950117

